# Effects of Isocyanate Structure on the Properties of Polyurethane: Synthesis, Performance, and Self-Healing Characteristics

**DOI:** 10.3390/polym16213045

**Published:** 2024-10-29

**Authors:** Hairui Wang, Lan Cao, Xiaolei Wang, Xiurui Lang, Wenwen Cong, Long Han, Hongyu Zhang, Huibin Zhou, Jujie Sun, Chengzhong Zong

**Affiliations:** School of Polymer Science and Engineering, Qingdao University of Science and Technology, Qingdao 266042, China; hrwang@mails.qust.edu.cn (H.W.);

**Keywords:** polyurethane, isocyanate, hydrogen bonding, adhesive, self-healing

## Abstract

Polyurethane (PU) plays a critical role in elastomers, adhesives, and self-healing materials. We selected the most commonly used aromatic isocyanates, 4,4′-methylene diphenyl diisocyanate (MDI) and tolylene-2,4-diisocyanate (TDI), and the most commonly used aliphatic isocyanates, hexamethylene diisocyanate (HDI), isophorone diisocyanate (IPDI), and dicyclohexylmethane-4,4′-diisocyanate (HMDI), as raw materials, combined with polytetramethylene ether glycol (PTMG) and 1,4-butanediol (BDO) to successfully synthesize five PU materials. The effects of isocyanate structure on polymerization rate, hydrogen bonding, thermal properties, phase separation, wettability, self-healing performance, adhesion, and mechanical properties were systematically investigated. The results show that isocyanates with higher symmetry facilitate hydrogen bonding, but excessive flexibility and crystallinity may inhibit its formation. MDI-based PU exhibits the highest hydrogen bonding index (HBI) of 4.10, along with the most distinct phase separation and the highest tensile strength of 23.4 MPa. HMDI-based PU demonstrates the best adhesion properties, with the highest lap shear strength of 7.9 MPa, and also exhibits excellent scratch healing ability. IPDI-based PU shows good self-healing performance, recovering 88.7% of its original tensile strength and 90.6% of its original lap shear strength after heating at 80 °C for 24 h. Furthermore, all the samples can be reprocessed by melt or solution methods, showing excellent recyclability.

## 1. Introduction

Polyurethane (PU) is one of the six most widely produced polymers globally, with a market size of USD 87.1 billion in 2023 [1,2]. PU is a multi-block copolymer composed of soft segment (SS) and hard segment (HS), where the SS is primarily formed by polymer polyols, and the HS is mainly composed of isocyanates and small molecule chain extenders. The HS typically contains a higher concentration of polar groups, which are the main contributors to the mechanical strength of PU, while the SS provides elasticity and flexibility at low temperatures [3,4,5]. PU exhibits advantages such as being lightweight, wear-resistant, chemically stable, biocompatible, mechanically robust, and highly customizable in terms of its chemical structure. These features make it widely applicable in elastomers, adhesives, foams, coatings, and other fields [6,7,8]. Inspired by nature, significant progress has been made in self-healing polymers, including polyurethane, over the past two decades. Self-healing materials, which can repair and partially restore their original functions either with or without external stimuli, enhance the durability and lifespan of materials. They find extensive applications in wearable devices, coatings, brain–machine interfaces, artificial skin, and flexible sensors [9,10,11]. Self-healing materials can be classified into intrinsic and extrinsic types. The construction of reversible dynamic bonds is a common strategy for developing intrinsic self-healing materials, with hydrogen bonding being one of the most prevalent reversible dynamic bonds. Hydrogen bonding is widely present in polyurethanes, making self-healing properties possible in these materials [12,13,14]. In polyurethanes, hydrogen bonds mainly occur between HS and HS, with fewer bonds forming between HS and SS. Therefore, the structure of the HS in PU plays a crucial role in the formation of hydrogen bonds, as well as in determining the mechanical and self-healing properties of the material.

Previous researchers have made continuous efforts to explore the relationship between the structure, composition, and content of HS and the macroscopic properties of PU. Yilgor et al. synthesized four chain extender-free PUs and used FTIR to study the effects of isocyanate symmetry on the microphase separation kinetics and thermodynamics of Pus [15]. Sáenz-Pérez et al. investigated the influence of 4,4′-methylene diphenyl diisocyanate (MDI) and tolylene-2,4-diisocyanate (TDI) on the thermal, shape memory, and mechanical behavior of PU, finding that shape memory behavior is dependent on hydrogen bond molecular interactions [16]. Similarly, Wang et al. developed dual-functional biopolyurethane blends with two different HS structures. By adjusting the ratio of isophorone diisocyanate (IPDI)-based PU to hexamethylene diisocyanate (HDI)-based PU, they modulated the stability of the hard domains in the blends, thus optimizing both the shape memory and self-healing properties of the PU [17]. In addition, the relationship between hard domains and the performance of polyurethanes has been explored in many studies [18,19,20]. MDI and TDI are the two most widely used aromatic isocyanates, while HDI, IPDI, and dicyclohexylmethane-4,4′-diisocyanate (HMDI) are the most commonly used aliphatic isocyanates. Therefore, these five isocyanates possess significant industrial value, and their structures vary widely, including both aliphatic and aromatic structures, with differing molecular symmetries and volumes. Consequently, studying the influence of isocyanate structure on polyurethane properties using these isocyanates as examples holds important theoretical and practical significance. However, few studies have collectively evaluated the effects of MDI, TDI, HDI, IPDI, and HMDI on the hydrogen bonding, thermal properties, phase separation, self-healing ability, adhesion, and mechanical properties of PU.

**Figure 1:**
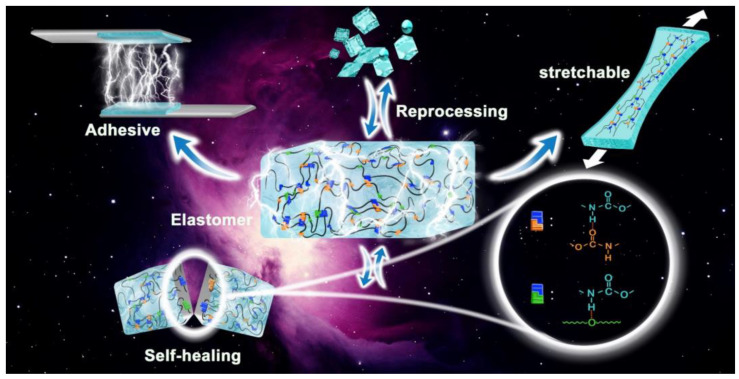
Schematic diagram of the properties and applications of polyurethane materials.

In this work, to better elucidate the relationship between the isocyanate structure and the performance of polyurethanes, we designed PU elastomers with a relatively high HS content. We maintained a molar ratio of isocyanate/PTMG/BDO = 3:1:2 and selected five commonly used isocyanates (HDI, TDI, MDI, HMDI, and IPDI) to systematically investigate the effects of the isocyanate structure on the hydrogen bonding, thermal properties, phase separation, self-healing ability, adhesion, and mechanical properties of PU. Additionally, the recyclability of the samples was assessed. This work further reveals the relationship between the isocyanate structure and performance, providing valuable insights into the structural design and material selection of PU in applications such as elastomers, adhesives, and self-healing materials (Figure 1).

## 2. Materials and Methods

### 2.1. Materials

Polytetramethylene ether glycol (PTMG, ≥99%, M_n_ = 1000 g/mol) was purchased from BASF Chemicals Co., Ltd., Shanghai, China. Hexamethylene diisocyanate (HDI, ≥99.5%), 4,4′-dicyclohexylmethane diisocyanate (HMDI, ≥99.5%), and 4,4′-methylene diphenyl diisocyanate (MDI, ≥99.6%) were purchased from Wanhua Chemical Group Co., Ltd., Yantai, China. Isophorone diisocyanate (IPDI, 99%), tolylene-2,4-diisocyanate (TDI, 95%), and dibutyltin dilaurate (DBTDL, 95%) were acquired from Shanghai Aladdin Biochemical Technology Co., Ltd., Shanghai, China. 1,4-butanediol (BDO, ≥99%) was purchased from Sinopharm Group Chemical Reagent Co., Ltd., Shanghai, China.

### 2.2. Preparation of PUs

**Figure 2:**
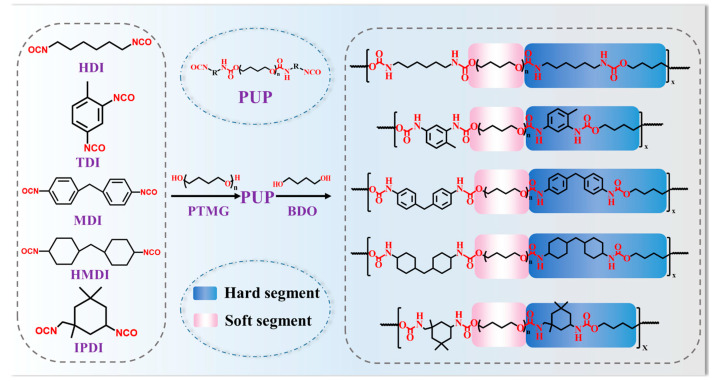
Synthesis mechanism schematic diagram of PUs.

PUs with different isocyanates were synthesized by the prepolymer method and named according to Table 1. PTMG was vacuum-dried at 120 °C for 2 h, then reacted with isocyanates at 80 °C under a N_2_ atmosphere for 2 h. The din-butylamine method determined the NCO group mass fraction (NCO%) in the polyurethane prepolymers (PUPs) to monitor the reaction degree [21]. BDO and DBTDL were added for the chain extension reaction and then hot-pressed at 100 °C for 30 min. Subsequently, the PUs were heated at 100 °C in an oven for 10 h. The synthesis mechanism of the PUs is illustrated in Figure 2. The HS content (weight percentage) of the PUs is denoted as Ch, calculated as Equation (1):

(1) $$Ch=\frac{m_{\mathrm{isocyanate}}+m_{\mathrm{BDO}}}{m_{\mathrm{isocyanate}}+m_{\mathrm{PTMG}}+m_{\mathrm{BDO}}}\times 100\%$$

where m_isocyanate_, m_PTMG_, and m_BDO_ are the masses of isocyanate, PTMG, and BDO, respectively.

### 2.3. Characterization

Fourier transform infrared spectroscopy (FTIR, VERTEX-70, BRUKER, Karlsruhe, German) was performed with 32 scans in the range of 4000–400 cm⁻¹ at a resolution of 4 cm⁻¹. Differential scanning calorimetry (DSC, DSC 3500, NETZSCH, Selb, Germany) was conducted under a nitrogen flow of 20 mL/min at a heating rate of 10 °C/min, with the temperature cooled from 25 °C to −100 °C, held for 2 min, then raised to 230 °C. X-ray diffraction (XRD, ULTIMA IV, RIGAKU, Tokyo, Japan) using Cu Kα radiation (λ = 0.15406 nm) was recorded from 5° to 80° at 5 °/min. A thermogravimetric analysis (TGA, TG209F3, NETZSCH, Selb, Germany) was carried out over a temperature range of 30–700 °C at 10 °C/min under a nitrogen flow of 20 mL/min. Dynamic mechanical analysis (DMA, Q800, TA, New Castle, DE, USA) was conducted from −100 °C to 100 °C at 3 °C/min in tensile mode. Surface morphologies were examined using atomic force microscopy (AFM, ICON, BRUKER) in the tapping mode. Water contact angle (WCA, JC2000D2, ZHONGCHEN, Shanghai, China) measurements were obtained at room temperature in the static mode. Tensile properties were tested using a universal testing machine (Z005, ZWICK, Ulm, Germany) according to ISO 37:2017, at 25 ± 2 °C and 50 ± 10% humidity, with a testing rate of 500 mm/min. A stereomicroscope was used to observe the scratch-healing behavior. The adhesive properties were assessed using a universal testing machine (AI-7000-M, Hirox, Tokyo, Japan) at a testing rate of 5 mm/min. Stainless steel substrates measuring 100 × 25 × 1.5 mm with a bonding surface length of 12.5 ± 0.25 mm were utilized for adhesive testing. The scratches were created using a surgical knife and healed at 80 °C. The mechanical properties of the samples after healing were tested under the same conditions as before. The healing efficiency (η) is defined as the ratio of the maximum recovered strength to the original strength, calculated according to Equation (2) [22,23,24].

(2) $$\mathrm{Healing}\;\mathrm{efficiency}\;\left(\eta \right)=\frac{\sigma_{\mathrm{heal}}}{\sigma_{\mathrm{pri}}}\times 100\%$$

where σ_heal_ is the tensile strength for healed specimens, and σ_pri_ is the tensile strength for the pristine specimens. The healing efficiency of lap shear strength was calculated using the same method.

## 3. Results and Discussion

### 3.1. Prepolymerization Reaction Rate and Structural Analysis of PUs

Due to the structural differences in isocyanates, the reactivity of NCO groups on their molecules varies. Figure S1 demonstrates the relationship between the NCO% and reaction time during the pre-polymerization of five different isocyanates with PTMG at 80 °C. The reaction between isocyanates and polyols is a nucleophilic addition reaction. It can be observed that due to the electron-withdrawing effect of aromatic rings and the electron-donating effect of aliphatic chains and rings, the reaction rates of TDI and MDI are significantly faster than those of HDI, HMDI, and IPDI. The reaction rate order is TDI > MDI > HDI > HMDI > IPDI.

**Figure 3:**
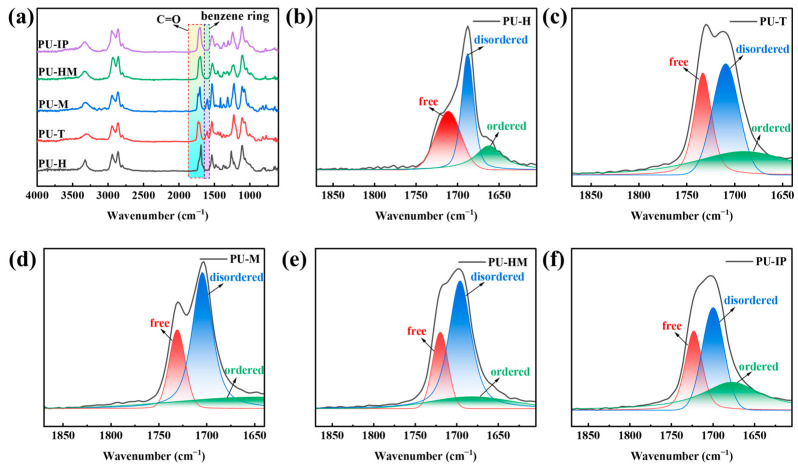
(**a**) FTIR full spectrum of PUs. (**b**) FTIR spectra in the C=O region of PU-H. (**c**) FTIR spectra in the C=O region of PU-T. (**d**) FTIR spectra in the C=O region of PU-M. (**e**) FTIR spectra in the C=O region of PU-HM. (**f**) FTIR spectra in the C=O region of PU-IP.

The chemical structures of the five PUs synthesized from different isocyanates were characterized using ATR-FTIR, as shown in Figure 3a. No characteristic peaks of -NCO groups were observed around 2260 cm^−1^ over the five PUs, indicating the complete conversion of isocyanate monomers to urethane groups [25,26]. The peaks at 3328 cm^−1^ and 1532 cm^−1^ represent the -N-H stretching and -N-H bending (amide Ⅱ), respectively [27,28]. The peaks at 2939 cm^−1^ and 2857 cm^−1^ belong to the antisymmetric and symmetric stretching vibration peaks of methylene (-CH_2_), respectively. PU-T and PU-M exhibited absorption peaks around 1600 cm^−1^, indicating benzene ring presence, while no peaks were observed at this position for the remaining samples, aligning with their respective molecular structures. The peak around 1104 cm^−1^ is associated with the stretching vibration of -C-O-C- [29]. The stretching vibration peak of C=O is approximately in the region of 1870–1605 cm^−1^ [30,31]. Due to the presence of hydrogen bonds between C=O and -N-H, the absorption peak of C=O can be divided into three peaks [32,33,34]. The peak around 1730 cm^−1^ is considered to be the free C=O peak, the peak around 1690 cm^−1^ corresponds to the disordered hydrogen-bonded C=O peak, and the peak around 1670 cm^−1^ is attributed to be the ordered hydrogen-bonded C=O peak. The absorption peak of C=O can be fitted by the Lorentz + Gauss function, and the results are shown in Figure 3b–f. The hydrogen bonding index (HBI) is defined as the ratio of the hydrogen-bonded carbonyl peak area to the free carbonyl peak area, indicating the degree of hydrogen bonding of materials. HBI is calculated by Equation (3) [21,29,35]:

(3) $$\mathrm{HBI}=\frac{A_{H-\mathrm{bond}C=O}}{A_{\mathrm{free}C=O}}$$

where A_H-bond C=O_ and A_free C=O_ represent the areas of the hydrogen-bonded and free C=O peaks.

The hydrogen bonding in PUs primarily arises from two sources: interactions between the -N-H and C=O groups, present between HSs; and interactions between the -N-H and -O- groups, found between HS and SS. The hydrogen bonding degree analysis result of C=O is shown in Table S1. Due to the lower symmetry and relatively low rigidity of the IPDI and TDI molecules, the molecular chains of PUs exhibit lower crystallinity and relatively good flexibility, facilitating the orderly arrangement of molecular chains to form ordered hydrogen bonding. Consequently, PU-IP and PU-T exhibit the highest proportion of ordered hydrogen bonding. HDI, MDI, and HMDI possess relatively good symmetry. In theory, HDI molecules have the best flexibility, minimal steric hindrance, and are most prone to forming hydrogen bonding. However, the excellent flexibility of PU-H leads to crystallization, thereby restraining the movement of molecular chains and hindering hydrogen bonding formation. Moreover, the greater molecular rigidity of MDI and HMDI also impedes hydrogen bonding formation to a certain extent. Additionally, HMDI, with a larger volume and greater steric hindrance compared to MDI, exhibits the lowest degree of ordered hydrogen bonding. Consequently, the final ranking of ordered hydrogen bonding involving the carbonyl groups in the five types of PUs is PU-IP ≈ P-T > PU-M > PU-H > PU-HM. Regarding the overall hydrogen bonding degree of the carbonyl groups, HDI, MDI, and HMDI exhibit good molecular symmetry, facilitating the proximity of molecular chains to form hydrogen bonding. However, due to the higher crystallinity of PU-H, its hydrogen bonding degree is the lowest. Conversely, the IPDI and TDI molecules, with lower symmetry and certain molecular volumes, exhibit lower hydrogen bonding degrees compared to PU-M and PU-HM. Therefore, the ranking of HBI for PU carbonyl groups is PU-M > PU-HM > PU-IP > PU-T > PU-H.

### 3.2. Thermal Behavior and Crystallization

**Figure 4:**
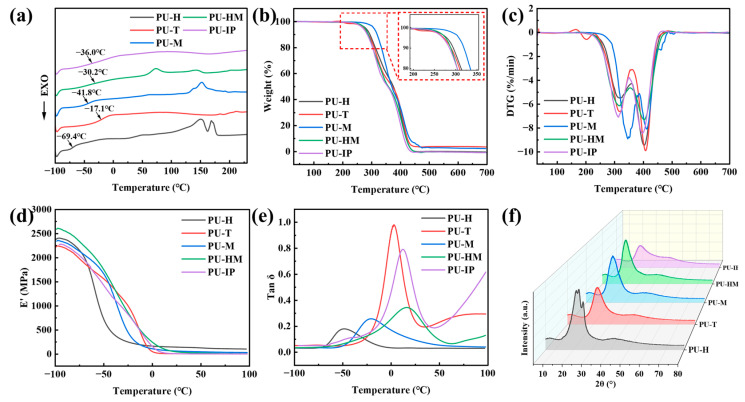
(**a**) DSC curves of PUs. (**b**) TGA curves of PUs. (**c**) DTG curves of PUs. (**d**) DMA curves of PUs. (**e**) Tan δ curves of PUs. (**f**) XRD patterns of PUs.

Figure 4a shows the DSC curves of the five PU samples. PU-H exhibits the best flexibility of molecular chains with the lowest internal rotation barrier, demonstrating the lowest glass transition temperature (T_g_) of −69.4 °C. Additionally, it exhibits the most prominent melting peak of HS crystallization at 150 °C and 169 °C. Due to the relatively good molecular symmetry of MDI, the HS is more concentrated, resulting in weaker constraints on the SS and more pronounced microphase separation [36]. Consequently, PU-M also shows a relatively low T_g_ of −41.8 °C, with a distinct crystallization melting peak near 152 °C. The larger molecular volume of HMDI affects the proximity between HS and the HS of PU, weakening the interaction between HS and HS, and increasing the interaction between SS and HS. Therefore, the T_g_ of PU-HM is higher than that of PU-M, while the degree of HS crystallization and the melting temperature of crystalline regions decreases. Due to the poorer molecular symmetry of IPDI and TDI [22], neither exhibits a prominent melting peak of HS crystallization in the high-temperature region. Moreover, due to the lower degree of HS aggregation and increased microphase mixing between SS and HS, the interaction between SS and HS to some extent restricts the movement of SS, resulting in higher T_g_ relative to PU-H and PU-M. Interestingly, PU-T shows the highest glass transition temperature of −17.1 °C.

The thermal stability of PUs plays a crucial role in practical applications, and it has been investigated using TGA. As shown in Figure 4b, all the samples exhibit similar thermal decomposition behavior with increasing temperature. The thermal decomposition of PUs can be mainly divided into two stages: the first stage (250–360 °C) primarily involves the decomposition of the HS, specifically the carbamate; while the second stage (360–500 °C) involves the decomposition of the SS, specifically the polyether chain [37,38]. Table S2 and the enlarged view in Figure 4b shows that PU-M has the highest initial decomposition temperature (T_5_, temperature at 5% weight loss) at 306 °C, mainly due to the presence of rigid phenyl rings in PU-M, which enhances the material’s heat resistance. In contrast, PU-IP exhibits the lowest initial decomposition temperature at 269 °C. Figure 4c presents the DTG curves of PUs, where the temperature corresponding to the fastest decomposition of the HS is denoted as T_h_ and that of the SS as T_s_. It can be seen that due to the different structures of the HS, the span of T_h_ varies significantly among the five PU materials, ranging from 315 °C to 346 °C. However, as PUs employ the same SS material, the fluctuation range of T_s_ is relatively small, ranging from 397 °C to 412 °C. Furthermore, the ordering of T_h_ and T_s_ is generally consistent, with PU-M exhibiting the highest T_h_ and T_s_ values at 346 °C and 412 °C, respectively.

The dynamic mechanical properties of PUs are illustrated in Figure 4d,e. Due to the larger molecular volume and relatively higher crystallinity of HMDI and MDI, PU-HM and PU-M exhibit comparatively higher storage modulus (E′) below −73 °C. PU-H, owing to its excellent crystallinity, also demonstrates an elevated storage modulus below −73 °C, falling between PU-HM and PU-M. Conversely, PU-IP and PU-T exhibit lower E′ at low temperatures due to relatively better flexibility and lower crystallinity. The peak temperature of the loss factor (tan δ) is typically considered as the material’s glass transition temperature (T_g_). Figure 4e reveals the T_g_ of PU-H, PU-T, PU-M, PU-HM, and PU-IP to be −48.2 °C, 3.0 °C, −20.7 °C, 15.8 °C, and 12.5 °C, respectively, which are basically in agreement with the DSC-derived sequence. The higher symmetry of HDI, MDI, and HMDI leads to the increased aggregation of HS, enhanced crystallinity, and higher phase separation degree. Consequently, during the glass transition, there is minimal intramolecular friction among the molecular chains, resulting in a lower tan δ (<0.4) for PU-H, PU-M, and PU-HM. Conversely, the lower symmetry of the TDI and IPDI molecules results in the reduced aggregation and crystallinity of HS, leading to higher intramolecular friction and higher tan δ during the glass transition. In the temperature range above T_g_ and below 100 °C, a consistent trend is observed in the values of the tan δ. PU-H and PU-M display low tan δ values (<0.1) due to their superior symmetry and crystallinity. PU-HM, with slightly lower symmetry and crystallinity, exhibits slightly higher tan δ. Conversely, PU-T and PU-IP, with the poorest symmetry and minimal HS crystallization, demonstrate higher tan δ values.

The crystalline properties of PUs were investigated via X-ray diffraction (XRD), with the results illustrated in Figure 4f. PU-T, PU-M, PU-HM, and PU-IP exhibited similar peak positions and profiles, characterized by a relatively strong peak at 2θ = 20–23° and a weaker peak near 2θ = 43°. The difference is that the peaks of PU-T and PU-IP at 20–23° are slightly weaker, indicative of typical amorphous characteristics, while those of PU-M and PU-HM are slightly stronger, indicating a certain degree of crystallinity. Notably, PU-H displayed three distinct peaks between 20 and 26°, with sharper profiles compared to the other PUs, indicating a higher degree of crystallization. At 2θ = 43°, PU-H resembled the other four PUs, showing a broad, weak peak characteristic of an amorphous phase.

### 3.3. Morphology, Wettability, Mechanical Property, and Self-Healing Ability

**Figure 5:**
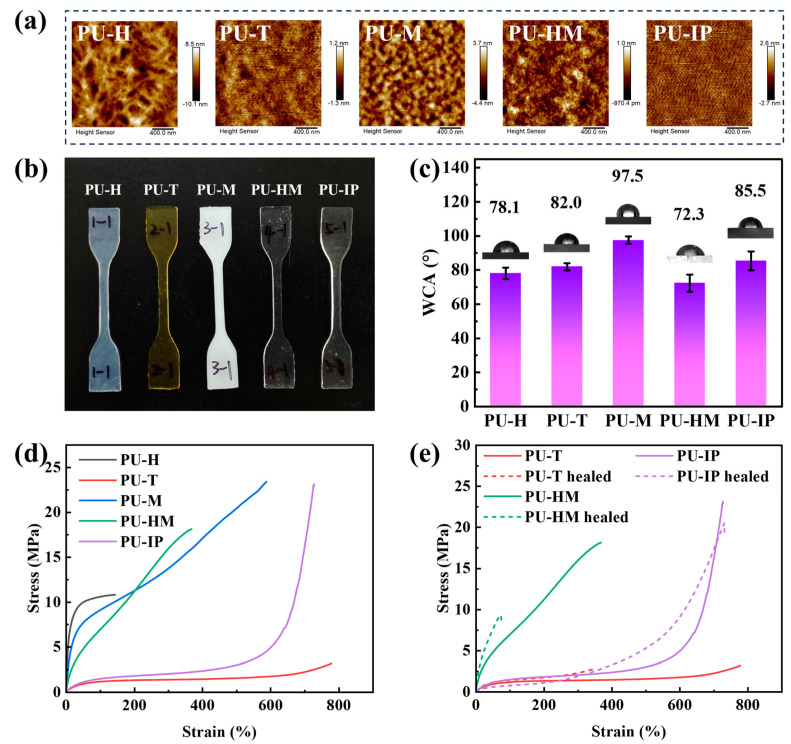
(**a**) AFM images of PUs. (**b**) Optical photo of dumbbell PU samples. (**c**) Water contact angles (WCA) of PUs. (**d**) Strain–stress curves of PUs. (**e**) Strain–stress curves of PU-T, PU-HM, and PU-IP after healing.

Atomic force microscopy (AFM) is a crucial tool for investigating the microphase separation behavior of PU. Figure 5a displays the AFM height images of the five PUs. In the figure, the bright regions represent the HS phase, while the dark regions represent the SS phase. It can be observed that the interface between the bright and dark regions in PU-M is distinct, with a uniform and fine distribution of SS and HS, indicating a clear microphase separation. This is mainly attributed to the presence of rigid aromatic rings in the HS phase of PU-M, along with the good symmetry of MDI, strong interactions between HS, and relatively good crystallinity, resulting in a more concentrated HS and more pronounced phase separation. In the AFM image of PU-HM, the interface between the bright and dark regions is somewhat blurred, indicating a slightly lower degree of phase separation compared to PU-M. The larger bright regions in PU-H are mainly attributed to its higher crystallinity. On the other hand, PU-T and PU-IP exhibit the lowest degree of microphase separation, which is attributed to the poorer molecular symmetry of TDI and IPDI and a loose accumulation of HS, leading to more interactions between SS and HS, promoting microphase mixing.

Figure 5b displays the optical photographs of the five PUs. PU-T, PU-HM, and PU-IP exhibit good transparency, PU-H appears semi-transparent, while PU-M is opaque and white. The varying transparency of the PUs is primarily related to the material’s crystallinity and crystal size. Due to the birefringence of crystals and the different refractive indices between the crystalline and amorphous regions, light cannot directly pass through the crystalline regions when the crystal size exceeds the wavelength of the incident light, resulting in refraction and reflection, thus reducing transparency. However, when the crystal size is smaller than the wavelength of the incident light, even with crystallinity present, it may not necessarily affect the transparency of PU. The yellow appearance of PU-T is primarily attributed to the presence of aromatic rings in its molecular structure, which may lead to the formation of quinone groups during sample preparation.

PU materials are widely used in car covers, waterproof and anti-fouling coatings, and artificial skin. Consequently, the hydrophilic and hydrophobic properties of their surfaces significantly impact their applicability in these areas. Contact angle (CA) is an important measure for evaluating wettability, primarily influenced by the surface energy and surface roughness of the solid. Figure 5c and Table S3 present the water contact angle (WCA) test results of the five PUs. PU-M exhibits the highest WCA of 97.5°, indicating hydrophobic characteristics. This could be attributed to the presence of numerous hydrophobic benzene rings in the main chain of PU-M molecules, along with its highest degree of hydrogen bonding, where polar groups mostly form hydrogen bonds within the molecules, limiting interactions with water molecules. The WCAs of PU-H, PU-T, and PU-IP are 78.1°, 82.0°, and 85.5°, respectively, consistent with the order of hydrogen bonding degrees. This indicates that when PUs have a higher degree of hydrogen bonding, polar groups tend to form hydrogen bonds internally, reducing surface energy and decreasing interaction with external water molecules, resulting in reduced hydrophilicity. However, PU-HM exhibits the smallest contact angle of 72.3° among these five PUs, indicating the highest hydrophilicity. This may be attributed to the minimal proportion of ordered hydrogen bonds in PU-HM, allowing for interactions between disordered hydrogen bonds and free polar groups with water molecules. Additionally, due to the larger molecular volume of HMDI, the molecular stacking of PU-HM is relatively loose, resulting in higher surface roughness of the final product, thus enhancing the material’s hydrophilicity.

Figure 5d shows the stress–strain curves of the PUs. The abundant hydrogen bonding within the HS promotes aggregation and crystallization, serving as the primary sources of strength via physical crosslinking points. Combining Figure 5d and Table S4 reveals that PU-M, with the highest degree of hydrogen bonding and superior crystallization, as well as rigid phenyl rings, exhibits the highest tensile strength of 23.4 MPa. PU-HM, structurally similar to PU-M but with slightly lower hydrogen bonding, also demonstrates relatively high tensile strength at 18.1 MPa. PU-H, possessing the lowest hydrogen bonding but excellent crystallization, displays the highest modulus and hardness. Additionally, the crystalline regions act as physical crosslinking points, enhancing strength to some extent, with a tensile strength of 10.8 MPa. However, extensive crystallization reduces material toughness, resulting in an elongation at break of only 144%. PU-T, due to its poor molecular symmetry, lower HS aggregation, and hydrogen bonding, exhibits a modest tensile strength of 3.2 MPa but boasts a remarkable elongation at break of 779%, the highest among the five PUs. Interestingly, PU-IP, despite having intermediate overall hydrogen bonding among these PUs, demonstrates an excellent tensile strength of 23.1 MPa and a fracture elongation of 728%, possibly attributed to its highest degree of ordered hydrogen bonding.

Figure 5e illustrates the stress–strain curves of the PU materials after healing at 80 °C × 24 h. During the experiment, PU-H and PU-M did not exhibit any significant mechanical property restoration, possibly due to their higher crystallinity and molecular rigidity, which hindered the diffusion and interaction of molecular chains between the fracture surfaces at 80 °C. PU-IP and PU-T demonstrated high healing efficiencies of 88.7% and 87.5%, respectively, whereas PU-HM exhibited a healing efficiency of 51.9%. This suggests that high crystallinity and molecular chain rigidity are detrimental to self-healing performance, while relatively lower crystallinity and loosely packed molecular chains are more conducive to the diffusion of molecular chains and the formation of interactions such as hydrogen bonding, resulting in improved healing effects.

### 3.4. Surface Scratch-Healing Properties

**Figure 6:**
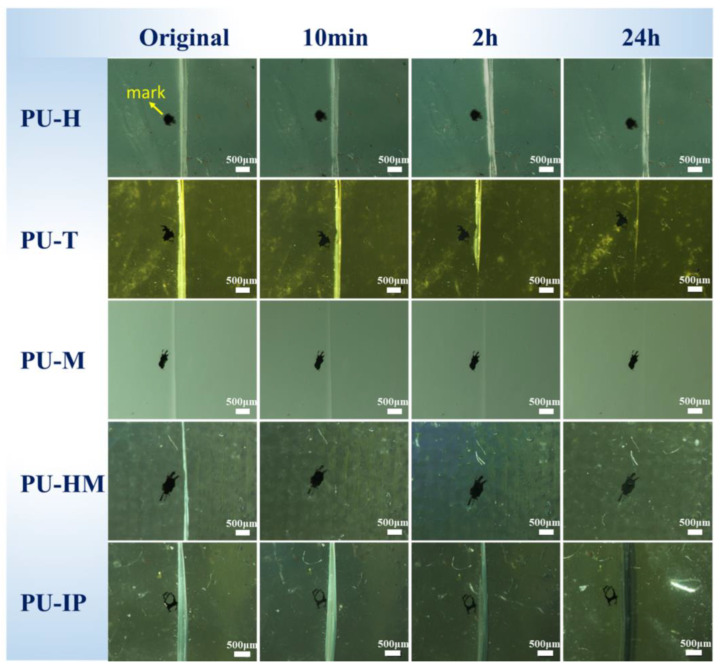
Optical microscopy photos of the scratch-healing process for the PUs.

To characterize the scratch-healing performance of the materials, the PUs were subjected to surface scratching using a surgical blade, with marks placed adjacent to the scratches. Subsequently, the samples were placed in an 80 °C oven for healing and removed at different time intervals for observation. Figure 6 presents the optical microscope images depicting the scratch-healing process of the PUs. PU-H and PU-M showed little to no evidence of scratch-healing, consistent with the results of the mechanical performance healing tests. Conversely, the scratches of PU-T and PU-IP gradually blurred at 80 °C, achieving relatively favorable healing outcomes after 24 h. Notably, PU-HM demonstrated remarkable scratch-healing effects, with the scratches being almost completely healed within just 10 min.

### 3.5. Adhesion Properties

**Figure 7:**
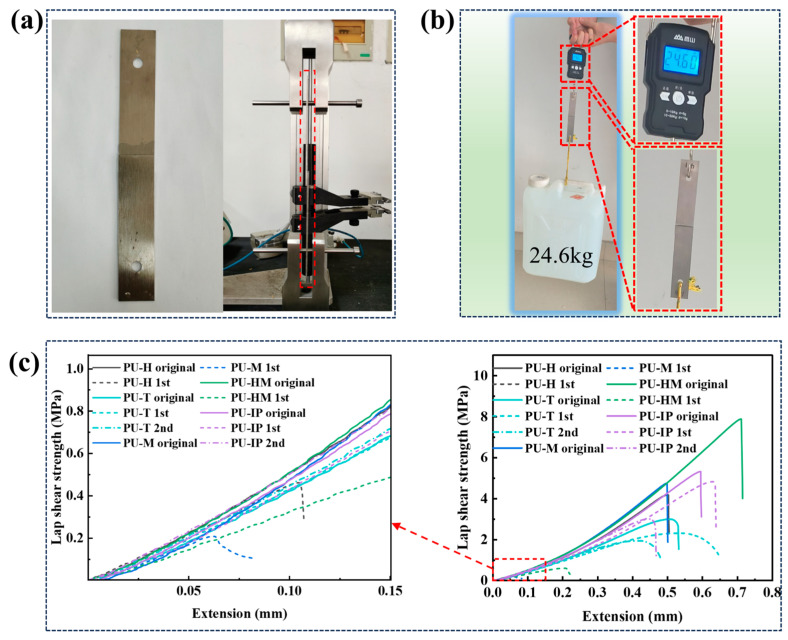
(**a**) Photos of the bonded steel plates and lap shear test. (**b**) The photo of the steel plates after PU-HM bonding lifting a 24.6 kg weight. (**c**) The initial and healed (80 °C × 24 h) lap shear test curves and enlarged images of the PUs.

After mixing the PUP and chain extender, the mixture was coated onto stainless steel plates, which were then overlapped and subjected to a 2 kg weight on top. The assembly was cured at 80 °C for 24 h and subsequently tested for lap shear strength using a universal testing machine. The photographs of the bonded steel plates and the lap shear test are shown in Figure 7a. Figure 7b illustrates, using PU-HM as an example, that the bonded steel plates could support at least 24.6 kg weight post-bonding. To evaluate the self-healing properties of the PUs as adhesives, the tested steel plates were reassembled after the lap shear test, a 2 kg weight was applied on top, and the assembly was subjected to another 80 °C curing for 24 h before retesting. This process was repeated for a second test, with the results presented in Figure 7c and Table S5. During curing, PU reacts with trace amounts of water adsorbed on steel plates and hydrated metal oxide to form urea. Urea groups (-NHCONH) and urethane groups (-NHCOO) in PU can form hydrogen bonds with metal oxide, while carbonyl groups and other electron-donating groups in PU can form coordinate bonds with iron and other metal atoms, enhancing the bonding strength. PU-HM exhibited the highest lap shear strength at 7.9 MPa, possibly due to its higher overall hydrogen bonding strength, providing greater mechanical strength, and a higher proportion of disordered hydrogen bonds, which can interact with the steel plates to enhance bonding strength. PU-H, PU-M, and PU-T showed no significant self-healing bonding effects, while PU-IP and PU-T exhibited healing efficiencies of 90.6% and 76.7% after the first healing, respectively, retaining 56.6% and 63.3% healing efficiencies after two healing cycles.

### 3.6. Recyclability and Reprocessability

**Figure 8:**
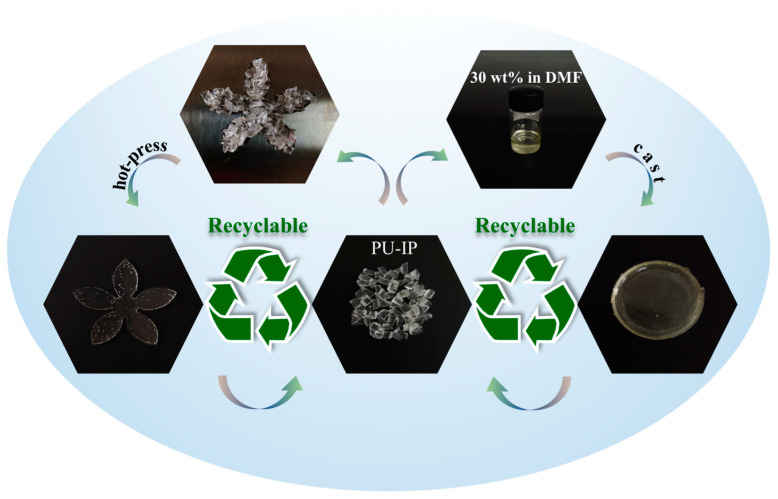
Schematic diagram of PU recycling and reprocessing via solution casting and hot pressing.

The molecular structure of the designed PU materials is linear and theoretically capable of melting and dissolving. However, this is not absolute and depends on the specific chemical composition and molecular structure. Therefore, the recyclability and reprocessability of the PU materials were assessed. Figure 8 illustrates two methods for reprocessing the PU materials. One involves cutting the PU materials into pellets, drying them at 100 °C for 2 h, and then placing them in a mold for hot pressing above the PU melting point and cooling to obtain the final product. The “petal” shape obtained from hot pressing demonstrates the material’s good flowability in the mold. The other method involves dissolving the PU materials in DMF, then pouring them into a mold and drying them to remove the solvent, resulting in the final product. The experimental results show that at a concentration of 30 wt%, the five PU materials dissolve slowly or are difficult to dissolve in DMF at room temperature but can dissolve within 24 h when heated to 80 °C. Particularly, PU-H, due to its excellent crystallinity, recrystallizes upon cooling to room temperature after dissolving in DMF at 80 °C, preventing the solution from flowing. Additionally, all five PU materials exhibit excellent melt-processing properties. The PU materials demonstrated good hot-press molding and solvent casting capabilities after two cycles of reprocessing validation.

## 4. Conclusions

In conclusion, we selected PTMG as the polyol and BDO as the chain extender, and using HDI, TDI, MDI, HMDI, and IPDI as the isocyanates, successfully synthesized a series of linear PUs with a relatively high HS content. The influence of the isocyanate structure on the hydrogen bonding degree, thermal properties, crystallinity, microphase separation, hydrophilicity, mechanical properties, self-healing, and adhesive properties of the PU materials was investigated. The results indicate that due to the good flexibility and molecular symmetry of HDI, PU-H exhibits the lowest T_g_ (−69.4 °C, detected by DSC) and the best crystallinity, leading to the highest hardness and modulus. PU-M demonstrates the highest HBI (4.10), contact angle (97.5°, the only one showing hydrophobicity), microphase separation, and maximum tensile strength (23.4 MPa). PU-HM has the highest lap shear strength, the smallest contact angle (72.3°), and the fastest scratch healing rate, with scratches healing almost completely within 10 min at 80 °C. PU-T has the largest elongation at break (779%). PU-IP exhibits relatively high tensile strength (23.1 MPa) and elongation at break (728%), as well as the highest healing efficiency (88.7%). All five materials can be recycled and reprocessed in both melt and solution forms. This study further elucidates the relationship between the isocyanate structure and polyurethane properties, providing guidance for researchers in the selection of materials when designing PU elastomers, adhesives, and self-healing materials.

## Figures and Tables

**Table 1:** The chemical composition of the PUs.

Sample	Isocyanate	Polyol	Chain Extender	Molar Ratio (Isocyanate/PTMG/BDO)	Molecular Weight of Isocyanate (g/mol)	Ch (%)
PU-H	HDI	PTMG	BDO	3:1:2	168.19	41
PU-T	TDI	PTMG	BDO	3:1:2	174.16	41
PU-M	MDI	PTMG	BDO	3:1:2	250.26	48
PU-HM	HMDI	PTMG	BDO	3:1:2	262.35	49
PU-IP	IPDI	PTMG	BDO	3:1:2	222.29	46

## Data Availability

The original contributions presented in the study are included in the article/Supplementary Materials, further inquiries can be directed to the corresponding author.

## Supplementary Materials

The following supporting information can be downloaded at https://www.mdpi.com/article/10.3390/polym16213045/s1, Figure S1: The change in NCO content with time during prepolymerization; Table S1: PU hydrogen bonding degree analysis; Table S2: PU thermal decomposition temperature analysis; Table S3: Contact angle values of the PUs. Table S4: Mechanical properties of PUs; Table S5: Lap shear properties of PUs.
