# Supplementary material for: Effects of Isocyanate Structure on the Properties of Polyurethane: Synthesis, Performance, and Self-Healing Characteristics
Source: Polymers (Basel). 2024 Oct 29;16(21):3045. doi: 10.3390/polym16213045 (PMC11548432; doi:10.3390/polym16213045)
Supplement: Supplementary file 1 [file polymers-16-03045-s001.zip › polymers-3280656-supplementary.pdf]

polymers

MDPI

Article

Effects of Isocyanate Structure on the Properties of Polyurethane: Synthesis, Performance, and Self-Healing Characteristics

Hairui Wang, Lan Cao, Xiaolei Wang, Xiurui Lang, Wenwen Cong, Long Han, Hongyu Zhang, Huibin Zhou, Jujie Sun and Chengzhong Zong *

School of Polymer Science and Engineering, Qingdao University of Science and Technology, Qingdao 266042, China

* Correspondence: qdzcz@qust.edu.cn

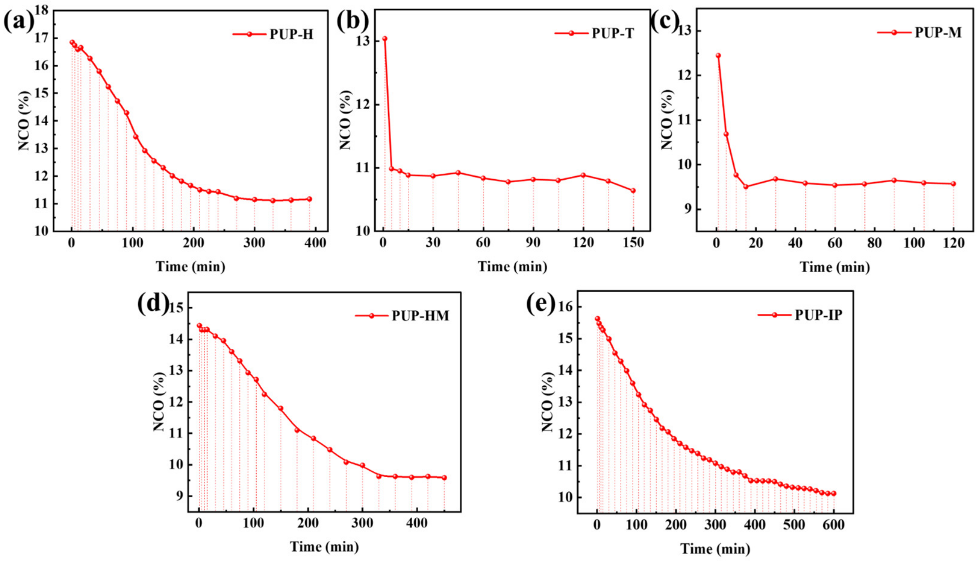

Figure S1. The change of NCO content with time during prepolymerization.

Table S1. PUs hydrogen bonding degree analysis.

	PU-H	PU-T	PU-M	PU-HM	PU-IP
Peak position of $C{=}O_{ordered\ H\text{-}bond}$ (cm^{-1})	1662.4	1690.1	1646.2	1682.0	1677.5
Proportion of $C{=}O_{ordered\ H\text{-}bond}$ (%)	23.7	36.7	27.7	16.9	39.8
Peak position of $C{=}O_{disordered\ H\text{-}bond}$ (cm^{-1})	1687.9	1709.2	1704.6	1696.0	1699.7
Proportion of $C{=}O_{disordered\ H\text{-}bond}$ (%)	40.6	36.6	52.8	62.5	35.3
Peak position of $C{=}O_{free}$ (cm^{-1})	1711.2	1733.1	1730.8	1720.0	1723.0
Proportion of $C{=}O_{free}$ (%)	35.7	26.7	19.5	20.7	24.9
Proportion of $C{=}O_{H\text{-}bond}$ (%)	64.2	73.3	80.5	79.3	75.1
HBI	1.80	2.75	4.10	3.83	3.02

Table S2. PUs thermal decomposition temperature analysis.

	PU-H	PU-T	PU-M	PU-HM	PU-IP
T_5/℃	279	272	306	276	269
T_h/℃	319	318	346	316	315
T_s/℃	403	408	412	403	397

Table S3. Contact angle values of the PUs.

Samples	CA (°)					
	1	2	3	4	5	Mean±SD
PU-H	79.5	79.6	72.2	79.8	79.4	78.1±3.3
PU-T	82.4	80.1	85.3	81.4	80.6	82.0±2.1
PU-M	95.6	95.6	96.7	99.4	100.3	97.5±2.2
PU-HM	67.9	71.4	67.4	76.7	78.2	72.3±5.0
PU-IP	82.6	90.0	77.7	86.0	91.0	85.5±5.5

Table S4. Mechanical properties of PUs.

Scheme 10.	Tensile strength (MPa)	Hardness (Shore A)	Tensile strength after healing (MPa)	Healing efficiency (%)
PU-H	10.8	93	--	--
PU-T	3.2	45	2.8	87.5
PU-M	23.4	93	--	--
PU-HM	18.1	83	9.4	51.9
PU-IP	23.1	53	20.5	88.7

Table S5. Lap shear properties of PUs.

Samples	Lap shear strength (MPa)	Lap shear strength after 1 time healing (MPa)	Healing efficiency after 1 time healing (%)	Lap shear strength after 2 times healing (MPa)	Healing efficiency after 2 times healing (%)
PU-H	4.2	0.5	11.9	--	--
PU-T	3.0	2.3	76.7	1.9	63.3
PU-M	4.7	0.2	4.3	--	--
PU-HM	7.9	0.6	7.6	--	--
PU-IP	5.3	4.8	90.6	3.0	56.6
